# Supplementary material for: CKAP2L Knockdown Exerts Antitumor Effects by Increasing miR-4496 in Glioblastoma Cell Lines
Source: Int J Mol Sci. 2020 Dec 27;22(1):197. doi: 10.3390/ijms22010197 (PMC7796349; doi:10.3390/ijms22010197)
Supplement: Supplementary file 1 [file ijms-22-00197-s001.zip › Supplementary materials (ijms-1047131)_proof-read/Figure S1. The Kaplan-Meier plot between high and low CKAP2L expression groups in different grading glioma.docx]

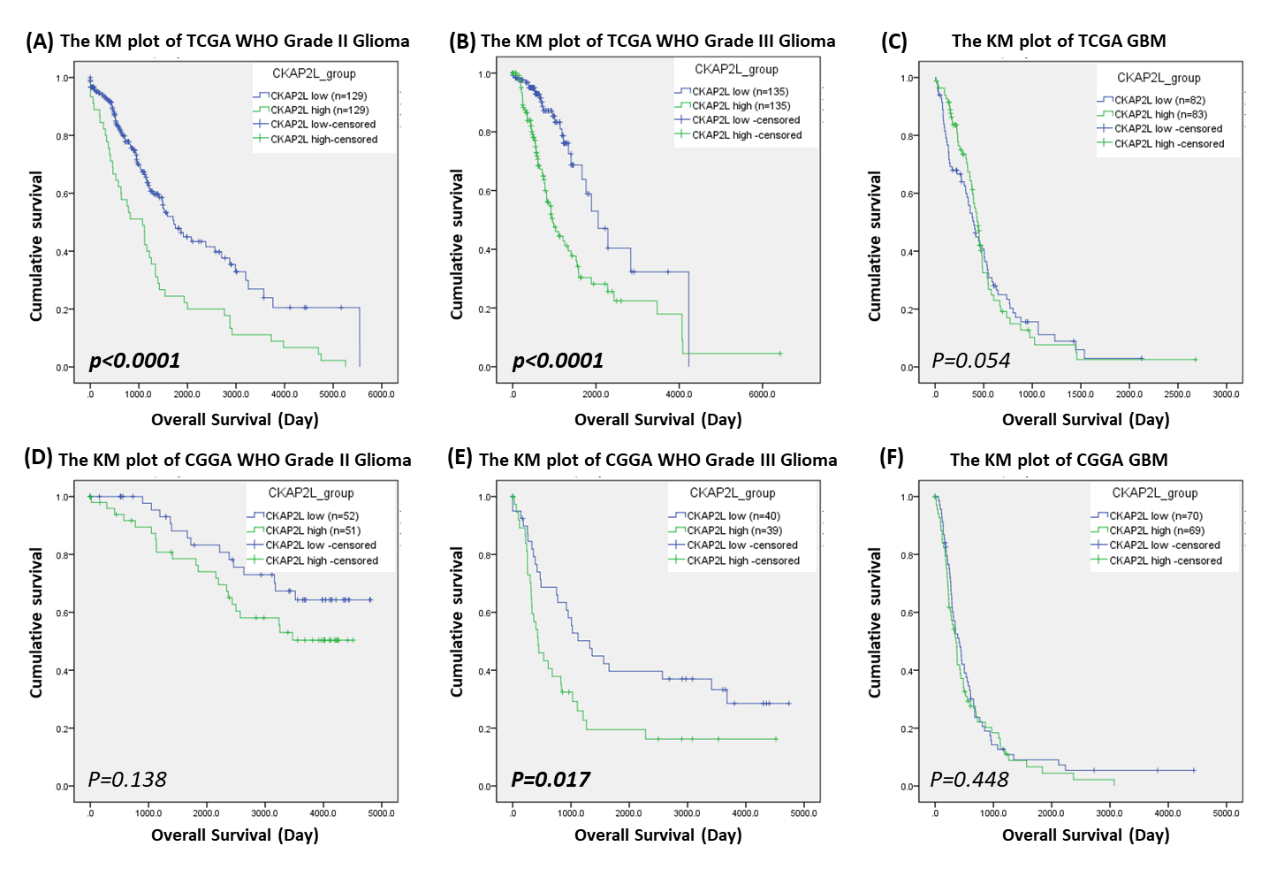


**Figure S1.** The Kaplan-Meier plot between high and low CKAP2L expression groups in each different grade. After further breakdown by the tumor grading, we found the grade III glioma revealed a survival difference between the high and low CKAP2L expression groups at TCGA and CGGA datasets (*p < 0.001* and *p = 0.017*, respectively). In the grade II gliomas, the curves revealed separation, but only TCGA data also reached statistical significance (*p < 0.001*). For the GBM clusters, the curves for high and low CKAP2L expression groups were closed and did not show differences statistically; however, the TCGA almost reached significance (*p = 0.054*).
