# Supplementary material for: CKAP2L Knockdown Exerts Antitumor Effects by Increasing miR-4496 in Glioblastoma Cell Lines
Source: Int J Mol Sci. 2020 Dec 27;22(1):197. doi: 10.3390/ijms22010197 (PMC7796349; doi:10.3390/ijms22010197)
Supplement: Supplementary file 1 [file ijms-22-00197-s001.zip › Supplementary materials (ijms-1047131)_proof-read/Figure S2. Compare the survival curve between TCGA and CGGA..docx]

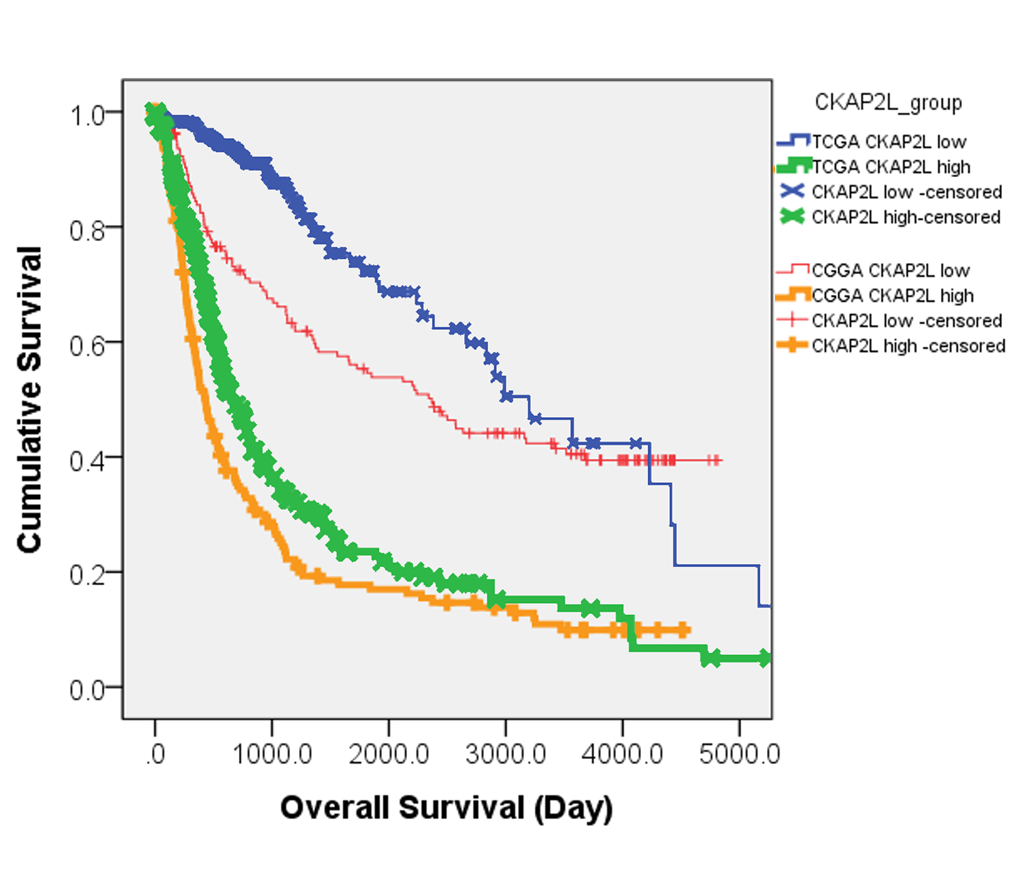


**Figure S2.** Compare the survival curve between TCGA and CGGA. From the comparison of both plots, we did notice that the overall survival of both low and high CKAP2L seems to be better in the CGGA, although the proportion of grade IV in CGGA is higher than TCGA. However, if we checked overall survival time before the 4000 days, both trends were similar or slightly better at the TCGA dataset.
