# Supplementary material for: CKAP2L Knockdown Exerts Antitumor Effects by Increasing miR-4496 in Glioblastoma Cell Lines
Source: Int J Mol Sci. 2020 Dec 27;22(1):197. doi: 10.3390/ijms22010197 (PMC7796349; doi:10.3390/ijms22010197)
Supplement: Supplementary file 1 [file ijms-22-00197-s001.zip › Supplementary materials (ijms-1047131)_proof-read/Figure S3. The Kaplan-Meier plot for high and low CKAP2L-score groups.docx]

**
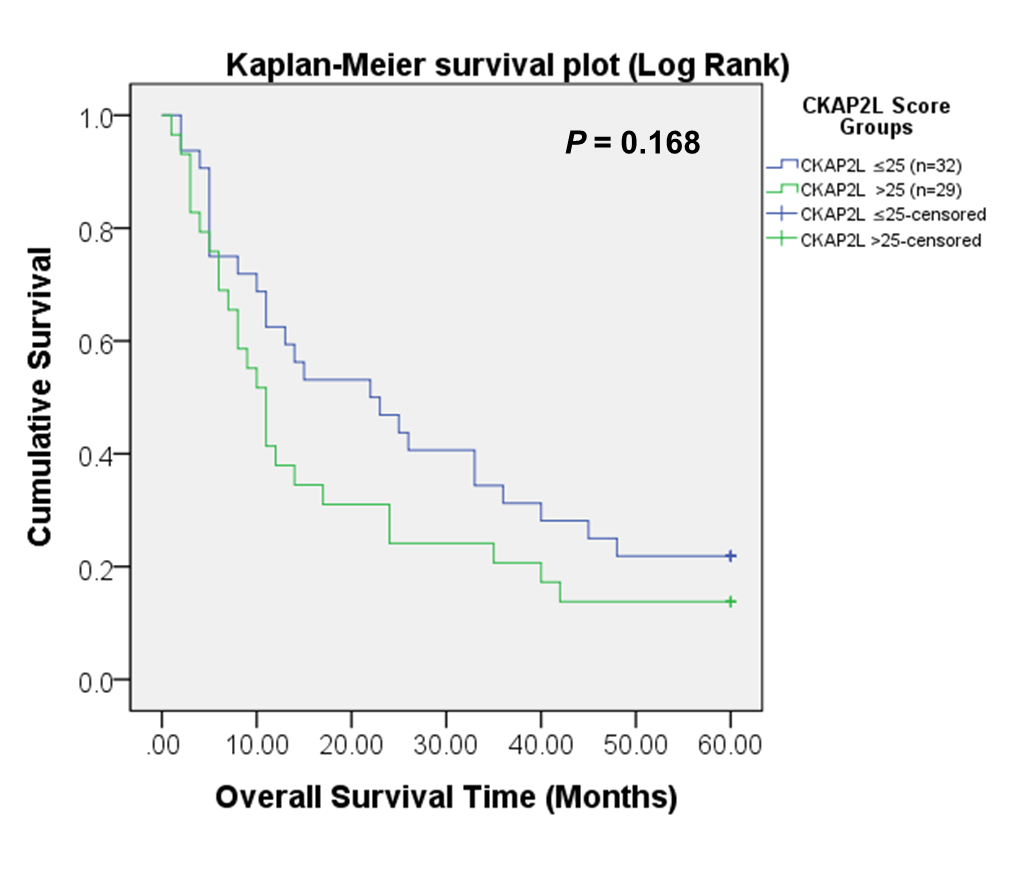
Figure 3.** The Kaplan-Meier survival plot for high and low CKAP2L-score groups by their median (CKAP2L-score ≤25 vs. CKAP2L-score>25). Here, we noticed that both plots were well separated, and the high CKAP2L-score seems to reveal a poorer prognosis. However, it did not reach statistical significance (*p* = 0.168).
