# Supplementary material for: CKAP2L Knockdown Exerts Antitumor Effects by Increasing miR-4496 in Glioblastoma Cell Lines
Source: Int J Mol Sci. 2020 Dec 27;22(1):197. doi: 10.3390/ijms22010197 (PMC7796349; doi:10.3390/ijms22010197)
Supplement: Supplementary file 1 [file ijms-22-00197-s001.zip › Supplementary materials (ijms-1047131)_proof-read/Table S4. Details of the antibodies used in this project.docx]

**Table 4.** Details of the antibodies used in this study.

| Antibody | Supplier | Product code | Dilution | Clonal type & Species |
| --- | --- | --- | --- | --- |
| CKAP2L | Sigma-Aldrich | HPA039407 | 1:100 | polyclonal rabbit |
| IDH1 (R132H) | Dianova | DIA-H09 | 1:100 | monoclonal mouse |
| ATRX | ATLAS | HPA001906 | 1:100 | polyclonal rabbit |
| H3K27M | Millipore | ABE419 | 1:100 | polyclonal rabbit |
| H3 K27me3 | Millipore | 07-449 | 1:100 | polyclonal rabbit |
| MGMT | Sigma | MAB-16200 | 1:50 | monoclonal mouse |
| EGFR | Millipore | 05-104-LA22 | 1:50 | monoclonal mouse |
| EGFRvIII | Absolute | Ab00184-L8A4 | 1:50 | monoclonal mouse |
| P53 | DAKO | DO-7 | 1:100 | monoclonal mouse |
| Neurofilament | DAKO | 2F11 | 1:100 | monoclonal mouse |
| NF1 | Abcam | HPA045502 | 1:100 | polyclonal rabbit |
| AxL | Sigma-Aldrich | HPA037423 | 1:50 | polyclonal rabbit |
| p-AxL | R&D system | Y779 | 1:50 | monoclonal mouse |
| NUR77 | Abcam | Ab217547 | 1:100 | polyclonal rabbit |
| PDGFRA | Santa Cruz | AF-307-SP | 1:100 | monoclonal mouse |
| GAPDH | Santa Cruz | sc-47724 | 1:10000 | monoclonal mouse |
| ACTN | Santa Cruz | sc-17829 | 1:10000 | monoclonal mouse |
| Cyclin D1 | Cell Signaling | 2978 | 1:500 | monoclonal rabbit |
| Cyclin B1 | Cell Signaling | 4135 | 1:1000 | monoclonal mouse |
| p-cdc2 | Cell Signaling | 9111 | 1:1000 | polyclonal rabbit |
| Cdc-2 | Cell Signaling | 77055 | 1:1000 | polyclonal rabbit |
| Beta-catenin | Santa Cruz | sc-7963 | 1:1000 | monoclonal mouse |
| E-cadherin | Cell Signaling | 3195 | 1:1000 | monoclonal rabbit |
| Twist | Santa Cruz | sc-81417 | 1:1000 | monoclonal mouse |
| Vimentin | Cell Signaling | 5741 | 1:5000 | monoclonal rabbit |
